# The epitranscriptome meets non-coding RNA: m6A-mediated regulation in oncogenesis and therapy

**DOI:** 10.3389/fcell.2025.1681555

**Published:** 2026-01-13

**Authors:** Prasanna Srinivasan Ramalingam, Mokhtar Rejili, Faouzi Haouala, Md Sadique Hussain, Yumna Khan, Mudasir Maqbool, Janaki Ramaiah Mekala, Sivakumar Arumugam

**Affiliations:** 1 Protein Engineering Lab, School of Biosciences and Technology, Vellore Institute of Technology, Vellore, Tamil Nadu, India; 2 Department of Biology, College of Sciences, Imam Mohammad Ibn Saud Islamic University (IMSIU), Riyadh, Saudi Arabia; 3 Uttaranchal Institute of Pharmaceutical Sciences, Uttaranchal University, Dehradun, Uttarakhand, India; 4 Independent Researcher, Abu Dhabi, United Arab Emirates; 5 Department of Pharmacology, Government Medical College Baramulla, Jammu and Kashmir, India; 6 Department of Integrative Biology, School of Biosciences and Technology, Vellore Institute of Technology, Vellore, Tamil Nadu, India

**Keywords:** epigenesis, oncology, RNA metabolism, RNA modifications, tumor microenvironment

## Abstract

The field of epitranscriptomics discovered N6-methyladenosine (m6A) modifications, which function as fundamental elements that control RNA metabolism properties that powerfully affect cancer biology. This review examines the way m6A modifications shape RNA stability while regulating translation, together with their eraser and reader proteins. We demonstrate that m6A modifications guide oncogene and tumor suppressor transcript outcomes, which promote tumor growth, metastasis, and therapeutic resistance. The regulatory function of m6A depends significantly on its relationship with ncRNAs that mainly include miRNAs, lncRNAs, and circRNAs. The review examines the effects of m6A on ncRNA production, stability, export, and degradation, as well as the regulation of m6A protein expression by ncRNAs, highlighting intricate reciprocal feedback loops that drive cancer progression. The interplay between m6A RNA modifications and ncRNAs provides emerging evidence on how they collectively influence the tumor microenvironment, modulate immune system responses, and contribute to resistance. Harnessing ncRNA-m6A interactions for managing drug resistance offers promising therapeutic avenues. However, advancing our understanding of the context-specific roles of m6A modifications and translating these insights into clinical applications remains a significant challenge. This review synthesizes recent findings on ncRNA-m6A crosstalk to lay the groundwork for developing epitranscriptomic strategies in precision oncology.

## 1 Introduction

The collective pool of chemical modifications known as epitranscriptome governs RNA fate and functional activity (Barbieri and Kouzarides, 2020). Among over 100 identified RNA modifications, N6-methyladenosine (m6A) is the most abundant internal modification in eukaryotic mRNA. m6A writes install this modification *via* the activity of a methyltransferase complex (METC) composed of Methyltransferase-like 3 (METTL3) and Methyltransferase-like 14 (METTL14), and associated partners such as Wilms’ tumor 1-associated protein (WTAP), Vir-Like m6A Methyltransferase Associated (VIRMA, also known as KIAA1429), and RNA-binding motif protein 15 (RBM15), while Fat Mass and Obesity-Associated Protein (FTO) and AlkB Homolog 5, RNA Demethylase (ALKBH5) act as erasers that remove m6A marks (Wang et al., 2020; Lou et al., 2024a). Reader proteins, including YTH-domain family members, insulin-like growth factor 2 mRNA-binding proteins (IGF2BP1/2/3), and heterogeneous nuclear ribonucleoproteins (HNRNP), bind to m6A marks to mediate subsequent RNA processing and translation regulation (Jiang et al., 2021). As a regulatory element in gene expression, m6A controls various post-transcriptional processes, including nuclear export, RNA stability, pre-mRNA splicing, and translation efficiency (Song et al., 2022; Yang C. et al., 2022).

Research indicates that the dysregulation of m6A writers and erasers is a fundamental driver of cancer development (Sun DY. et al., 2025). Tumorigenesis often involves aberrant expression patterns of m6A writers, erasers, and readers, leading to significant alterations in cancer epitranscriptome (Gao et al., 2021). The effects of m6A modifications in cancer cells operate through distinct mechanisms, depending on the specific cellular context. Oncogene transcripts exhibit increased stability, and pro-tumorigenic non-coding RNAs (ncRNAs) are more efficiently processed when m6A methylation levels are elevated, whereas the loss of m6A marks on tumor-suppressor mRNAs and lncRNAs prolongs their expression, promoting malignant phenotypes​ (Li Y. et al., 2024). Studies demonstrate that disruptions in the m6A pathway can either activate oncogenic signaling or suppress tumor growth, through such pathological alterations are frequently associated with poor clinical outcomes (Uddin et al., 2021). The discovery of m6A as an epigenetic RNA modification reveals its dual roles in cancer development, akin to DNA methylation (Feng et al., 2025), while offering novel opportunities to target m6A regulatory mechanisms in cancer therapy.

The class of regulatory RNAs known as ncRNAs comprises diverse elements that do not encode proteins but play significant roles in regulating gene expression (Yan J. et al., 2025). Classification systems categorize ncRNAs based on their features and functions, including short post-transcriptional regulators called microRNAs (miRNAs), long ncRNAs (lncRNAs) exceeding 200 nucleotides, and covalently closed circular RNAs (circRNAs) (Wu et al., 2025; Gong et al., 2024). Research demonstrates that ncRNAs can function either as oncogenes or tumor suppressors, depending on cellular context. Dysregulation of ncRNAs is frequently observed in various cancers, contributing to cancer hallmarks such as uncontrolled proliferation, apoptosis evasion, angiogenesis, invasion, and metastasis (Zhou L. et al., 2020). Cancer-associated miRNAs “oncomiRs,” typically silence tumor-suppressor transcripts, whereas specific miRNAs and lncRNAs act as anti-oncogenes by repressing proto-oncogene expression or preserving tumor-suppressive networks (Lin et al., 2019). For example, the metabolic activities of lncRNAs such as HOTAIR and MALAT1 are upregulated in tumors, promoting metastasis (Sun and Ma, 2019; Hussain et al., 2024), while the lncRNA MEG3 inhibits tumor growth (Hussain et al., 2023), highlighting the multifaceted roles of ncRNAs in cancer.

The clinical significance of ncRNAs, combined with their tissue-specific expression patterns, has prompted researchers to explore their potential as biomarkers and therapeutic targets in oncology. Many ncRNAs are detectable in accessible body fluids and tumor samples, making them promising candidates for diagnostic and prognostic biomarkers (Ebrahimi et al., 2022). The development of ncRNA-targeted therapies has advanced rapidly, with miRNA mimics and anti-miRNA oligonucleotides aiming to recover tumor-suppressor miRNAs and inhibit deregulated oncomiRs (Gambari et al., 2016). Similarly, preclinical studies show that the small interfering RNA (siRNA) and antisense oligonucleotides (ASOs) targeting oncogenic lncRNAs yield therapeutic benefits (Khan et al., 2017). RNA-based interventions continue progressing towards clinical trials, underscoring the therapeutic potential of ncRNA targeting in cancer (Sun M. et al., 2025). Additionally, the inherent stability of circRNAs and lncRNAs in circulation offers new opportunities for both drug delivery platforms and therapeutic targeting. Overall, ncRNA-based therapies present a novel approach that complements and enhances conventional protein-targeted cancer treatments.

m6A modifications have emerged as crucial epigenetic regulators of ncRNA activity and biogenesis during cancer development (Chen et al., 2022a). These modifications enhance the recognition of primary miRNA transcripts (pri-miRNAs) by the Microprocessor complex (Drosha/DGCR8), thereby increasing the production of mature miRNAs​ (Knuckles et al., 2017). Through this mechanism, m6A influences miRNA in cancer cells, leading to alterations in gene networks that control proliferation and apoptosis. For instance, METTL3-mediated m6A methylation enhances the processing of oncogenic pri-miRNAs, such as those in the miR-17–92 cluster, while the loss of m6A reduces the production of tumor-suppressor miRNAs (Mei et al., 2023).

The structural integrity and functional impacts of lncRNAs are also closely tied to m6A modifications. The presence of m6A residue alters local RNA structures, exposing or concealing regions critical for interaction with RNA-binding proteins. Specifically, m6A deposition on lncRNAs generates binding platforms for HNRNPC, facilitating lncRNA-protein interactions to modulate gene expression (Lin H. et al., 2022). Additionally, m6A modifications stabilize lncRNAs, enhancing their functions as competitive endogenous RNA (ceRNAs) that sequester miRNAs (Wen et al., 2023). This increased sequestration of tumor-suppressive miRNAs elevates the expression of oncogenic target genes. In colorectal cancer (CRC), for example, the pro-tumor activity of the lncRNA XIST is counteracted by METTL14-mediated m6A modification, which recruits the reader YTHDF2 to promote XIST degradation (Chen DH. et al., 2021). Similarly, m6A-modified circRNAs display dual functions: they can initiate peptide translation and serve as binding sites for m6A readers involved in oncogenic signaling. In squamous cell carcinoma, m6A-modified circMMP9 recruits IGF2BP2, forming a complex with the ETS1 transcription factor to activate oncogene expression (Barbieri and Kouzarides, 2020). The findings highlight the dynamic and context-dependent roles of m6A-ncRNAs interactions, which can either promote or inhibit tumor progression. In some cases, a single methyl group shift on an ncRNA may accelerate cancer cell growth, while in others it introduces vulnerability that suppresses malignancy, underscoring the complexity and therapeutic potential of this regulatory layer in cancer biology.

The integration of epitranscriptomics and ncRNAs research in cancer has established a novel interdisciplinary field with direct implications for therapeutic development. It is widely recognized within the scientific community that maximizing the benefits of RNA-based therapeutics requires a comprehensive understanding of epitranscriptomic mechanisms that regulate ncRNA function. The pharmaceutical industry’s growing interest in RNA modifications is exemplified by the development of METTL3 inhibitors for the treatment of acute myeloid leukemia (AML) (Tzelepis et al., 2019; Yankova et al., 2021). Rapid advances in ncRNA-directed diagnostics and therapeutics underscore significant progress, as numerous miRNA- and lncRNA-based agents are currently progressing through preclinical and clinical stages (Grillone et al., 2024).

Despite this momentum, critical gaps remain in understanding the interplay between m6A modifications and ncRNAs. Current research on m6A in ncRNAs lacks a comprehensive synthesis of its modulation effects on cancer initiation, tumor progression, and therapeutic response. Key questions persist regarding how to achieve selective control over m6A modifications and their ncRNA targets to optimize therapeutic efficacy while minimizing off-target effects.

The review aims to consolidate recent evidence on how cancer influences m6A epitranscriptomic modifications and ncRNAs dysregulation. We provide a comprehensive overview of the biological roles of m6A across various ncRNA classes and explore their functional interactions in promoting or suppressing tumorigenesis. By analyzing current findings and identifying open questions, we propose potential m6A–ncRNA–based intervention strategies for precision oncology. A deeper understanding of RNA modifications may enable the development of innovative therapies, whether through modulation of m6A writer/eraser or the design of RNA therapeutics that leverage epitranscriptomic marks to drive next-generation cancer treatments.

## 2 m6A modifications: writers, erasers, and readers

m6A RNA methylation installation, removal, and recognition with specific protein families commonly referred to as writers, erasers, and readers, respectively. These variables work collectively to dynamically control the destiny and purpose of the RNA, and their dysregulation can be able to reprogram the gene expression in cancer cells. m6A modification appears in most transcripts, and the ratio of m6A/A in mRNA ranges between 0.2% and 0.5% (Li Q. et al., 2020). Each class is covered below, with its molecular components, biological functions, and recent trends of associating that class with tumorigenesis, cancer progression, and treatment resistance. We start with the METC writers, the demethylase erasers, and the m6A-binding readers.

### 2.1 Writers/methyltransferases

m6A methylation is facilitated by a multicomponent METC (Bokar et al., 1994). METTL3, an S-adenosylmethionine (SAM)-binding protein, is the first identified constituent of the m6A METC and is extensively consistent among eukaryotes (Bokar et al., 1997). METTL14 constitutes an additional active element of the m6A METC. METTL3 and METTL14 integrate inside nuclear speckles and form a persistent heterocomplex at a 1:1 ratio (Liu et al., 2014; Wang P. et al., 2016). METTL3 predominantly acts as the catalytic center, whereas METTL14 provides a structural foundation for RNA association (Wang X. et al., 2016). WTAP constitutes an additional essential element of the m6A METC. WTAP does not possess a consistent catalytic methylation space, rendering it incapable of catalyzing m6A alteration. WTAP functions as an adapter protein that engages with both METTL3 and METTL14, substantially influencing the cellular RNA m6A burden (Liu et al., 2014). Furthermore, WTAP could engage with different proteins and lncRNAs (Horiuchi et al., 2013), indicating that WTAP may enlist more elements in the METC. Methyltransferase-like 16 (METTL16), a homolog of METTL3, is a recently discovered m6A METC that regulates cellular SAM levels and methylates U6 short nuclear RNA (Pendleton et al., 2017). Additionally, additional proteins, including VIRMA (Schwartz et al., 2014), RBM15 and its paralogue RBM15B (Patil et al., 2016), as well as ZC3H13 (Wen et al., 2018), have been identified as constituents of the m6A METC and are essential for m6A methylation. However, their roles vary markedly by tumor type, as discussed in Section 4.8.

Additionally, besides the internal m6A in vertebrate mRNAs, when the transcription begins nucleotide is adenosine, its N6 position undergoes methylation to become N6, 2′-O-dimethyladenosine (m6Am). A comprehensive transcriptome study demonstrated that N6-methylation of m6Am enhances the translation of capped mRNAs. Recent research discovered PCIF1, a cap-specific adenosine methyltransferase (CAPAM), to be accountable for the N6-methylation of m6Am (Akichika et al., 2019). Consequently, the METC for m6Am is unique from that of m6A.

### 2.2 Erasers/demethylases

Erasers are a category of proteins that remove the methyl groups from segments of RNA modified by m6A mutations. This encompasses two particular types of demethylating enzymes: FTO and ALKBH5. The inclusion of erasers enables m6A to experience dynamic and reversible modifications. FTO is the first identified m6A demethylase, located in nuclear speckles and the cytoplasm. Research indicates that FTO exhibits effective oxidative demethylation capabilities at multiple m6A locations in RNA (Jia et al., 2011). The FTO may also go after m6Am in mRNA and snRNA, in addition to m6A. This association is crucial for mRNA rigidity, as it aids in resisting DCP2-mediated mRNA decapping, as demonstrated by investigations (Wei et al., 2018; Mauer et al., 2017). The FTO primarily promotes the elimination of the methyl group from m6A in the nucleus, while also regulating the elimination of methyl groups from both m6A and m6Am in the cytoplasm. Unanimity exists regarding FTO’s priority for m6A *versus* m6Am; however, it is widely accepted that FTO exhibits an increased liking for m6A (Jia et al., 2011; Mauer et al., 2017).

ALKBH5 is a recognized protein that facilitates the removal of m6A methylation. Its influence on nuclear RNA breakdown, exportation, and gene regulation is extensive, with involvement in crucial steps through its demethylation activity in both *in vivo* and *in vitro* contexts. ALKBH5 demethylates single-stranded RNA fragments containing m6A, functioning similarly to FTO (Zheng et al., 2013). FTO and ALKBH5 belong to the iron and two-oxoglutarate-dependent family of AlkB oxygenases, yet they exhibit distinct biological functions. FTO is significantly linked to obesity, while ALKBH5 is essential for spermatogenesis (Zheng et al., 2013; Church et al., 2010). FTO exhibits elevated rates of expression in the brains of mice (Gao et al., 2010), whereas ALKBH5 demonstrates substantial expression in the testes (Hong et al., 2022). Both may cautiously initiate the demethylation of specific target mRNAs, resulting in varied biological implications.

### 2.3 Readers

Proteins that primarily bind to the methylation spot of m6A are termed readers. They recognize variations in m6A on targeted RNA and are involved in various RNA metabolic processes. Readers include YTHDC1-2 and YTHDF1-3 (YTH domain-containing proteins), IGF2BP1-3, and hnRNPA2B1 and HNRNPC/G (Xiao et al., 2016; Hsu et al., 2017; Shi et al., 2017). YTHDC1 engages with KDM3B, an enzyme that demethylates histone H3 at lysine 9. This connection takes place in chromatin regions associated with the chemical alteration known as m6A. The demethylation of H3K9me2 by KDM3B, aided by YTHDC1, leads to increased gene transcription (Li Y. et al., 2020). Conversely, YTHDC2 diminishes the longevity of m6A-modified mRNA through interaction with RNA helicase. It boosts the efficiency of converting peculiar mRNA (Tanabe et al., 2016; Wojtas et al., 2017; Mao et al., 2019).

Although YTHDF proteins possess a similar arrangement, their functions differ significantly. YTHDF1, YTHDF2, and YTHDF3 engage with specific mRNAs, regulating both the viability and translation of mRNAs linked to YTHDF proteins (Lan et al., 2021; Ries et al., 2019; Wang et al., 2014). YTHDF1 facilitates translation and improves the fidelity of m6A-tagged transcript translation, leading to elevated protein synthesis (Wang et al., 2015). The YTHDF2-mRNA complex comprises two structural domains: the C-terminal domain interacts with m6A-mRNA, whereas the N-terminal domain is crucial for the localization of the complex to cellular RNA destruction sites (Wang et al., 2014). YTHDF3 facilitates the production of proteins *via* YTHDF1 and increases the degradation of methylated mRNA *via* YTHDF2 (Shi et al., 2017). The three YTHDFs significantly influence key biological operations associated with m6A RNA methylation in an integrated way.

IGF2BPs promote RNA stabilization and increase mRNA retention in varying physiological conditions by recruiting RNA stabilizers such as matrin 3. RNA stabilizers include ELAV-like RNA-binding protein one and poly (A)-binding protein cytoplasmic 1 (Huang et al., 2018). hnRNPs play critical roles in regulating transcriptional and post-transcriptional processes (Wu et al., 2018). Alarcon et al. demonstrated that hnRNPA2B1 acts as a nucleic acid reader for the m6A alteration and facilitates the transcription of a specific subset of pri-miRNAs dependent on METTL3. hnRNPA2B1 is capable of binding to nuclear RNAs, including the G (m6A) C sequence, in both biological systems and experimental environments. The Drosha-DGCR8 complex can be assembled to protect RNA-targeted regions from degradation by ribonucleases (Alarcón et al., 2015). Li et al. proved that METTL3-induced m6A methylation of LINC01 833 promotes the progression of non-small cell lung cancer (NSCLC) by regulating hnRNPA2B1 (Li D. et al., 2022). Furthermore, research suggests that m6A may enhance the ability of hnRNPA2B1 to facilitate nuclear reactions such as the pri-miRNA process. This is accomplished by increasing the accessibility of hnRNPA2B1 to designated attachment zones, instead of connecting with m6A (Wu et al., 2018). Additional research is necessary to elucidate the intricacies of the m6A system. These functions are further compartmentalized and vary by tumor lineage; check the next section for a detailed comparison of nuclear *versus* cytoplasmic reader roles across cancer types.

Even though the domains of YTHDF proteins are highly similar, the recent functional dissection findings prove that YTHDF1 and YTHDF3 are not interchangeable translational activators. YTHDF1 is a translation enhancer specific to cap-dependent initiation which promotes the recruitment of eIF3 and eIF4G to activates oncogenic transcripts including MYC, SNAI1, and immune regulation mRNAs (Shirokikh and Preiss, 2018). YTHDF3 on the other hand acts as a co-activator and an amplifier of RNA decay (Shi et al., 2017), based on interaction partners. In breast cancer, it is the YTHDF3 that selectively stabilizes m6A-modified lncRNAs to drive EMT (Lin Y. et al., 2022), but in liver cancer it is the circRNA-encoded micropeptides that are preferentially promoted by YTHDF3 through m6A loading onto ribosomes (Yi et al., 2024). Therefore, YTHDF1 is more of a pro-translation protein, whereas YTHDF3 combines both translation and decay responses in a context-dependent and not a redundant paralog. Beyond the canonical YTH and IGF2BP families, recent work has highlighted FXR1 as a clinically relevant RBP that integrates oncogenic post-transcriptional programs. Mechanistic and *in vivo* studies show that FXR1 binds and destabilizes specific mRNAs such as PDZK1IP1 and ATOH8 to promote esophageal cancer progression, while broader transcriptomic analyses reveal its capacity to coordinate oncogene and tumor-suppressor networks across multiple cancer types (Khan et al., 2024a; Khan et al., 2024b). An updated review further summarizes the multifaceted roles of FXR1 in cancer and other diseases, situating it within the wider landscape of m^6^A-sensitive RBPs and post-transcriptional regulation (Khan et al., 2024c).

A second regulatory axis entails competition of shedding in the data transferred by YTHDF2 and the stabilization by IGF2BP/FXR1. In AML and HCC, the YTHDF2 scaffolds CCR4-NOT and HRSP12/MRP endonuclease complexes, which stimulate the rapid turnover of tumor-suppressive transcripts (Zhong et al., 2019). Nevertheless, at microenvironmental stress, like hypoxia, YTHDF2 may alter stabilizing genes of survival functions, including EGFR, demonstrating its non-canonical action in some tumors (Xu P. et al., 2022). On the other hand, stabilizing readers are IGF2BPs (1/2/3) and FXR1 which stabilize oncogenic mRNAs by protecting against degradation through shielding of m6A-marked locations and binding HuR or Matrin-3 (Khan et al., 2024b; Sun et al., 2022). This leaves behind a tug-of-war where the most powerful reader, YTHDF2 (decay) and IGF2BP/FXR1 (stabilization) either causes an m6A-modified transcript to be quickly destroyed or stayed stabilized. Balance between these pathways is different in tumor types, cell stress, and levels of particular ncRNAs, and hence there is a need to read m6A phenotypes in a tumor-specific molecular context.

In all feasible cases, mechanistic roles of m6A in interactions with other entities should be based on structural or biophysical validation. A few of the above-described interactions, including YTHDF1/2/3 recognition of the m6A consensus, YTHDC1 has been experienced-based on the agreement between YTHDC1 and H3K9me2 chromatin and hnRNPA2B1-mediated pri-miRNA processing are upheld by CLIP-seq or RIP-seq data demonstrating direct relationship with the experiment. The interface between YTH domain and m6A has also been resolved by cryo-EM and crystallographic methods, which show the recognition of aromatic cages in response to the docking of peptides and the stabilization of the conformation, which can be used as a structural factor to explain reader specificity (Xu et al., 2014; Su et al., 2022). Nevertheless, most of the associations with ncRNAs are still of a correlational nature without being backed up by crosslink-based mapping or high-resolution structural analysis. Additional studies in the future incorporating CLIP-seq, SHAPE-MAP and structural analyses will be necessary to uncover the difference between direct interactions and indirect regulatory responses.

### 2.4 Context-specific roles of m6A readers across cellular compartments and tumor types

The recent findings indicate that m6A reader proteins provide highly compartmentalized and cancer-type-specific functions, but it does not necessarily mean that they behave as equally oncogenic or tumor-suppressive regulators as traditionally thought. Rather, their biological behavior is determined by their localization within the cell and their interaction with other proteins, and the molecular landscape of specific types of tumors. RNA splicing, chromatin regulations, and RNA export organization are mostly activities controlled by nuclear m6A readers, but the outcomes of these mechanisms vary significantly in the cancers (Tingey et al., 2022). As a case in point, in breast cancer, YTHDC1 upregulates alternative splicing of oncogenic transcripts but in hepatocellular carcinoma (HCC) biogenesis of tumor-suppressive circRNAs occurs (Luxton et al., 2019). Equally, the hnRNPA2B1, a reader that is involved in pri-miRNA processing, promotes the metastatic program in lung adenocarcinoma (LUAD); however, it promotes the immune activation pathways in melanoma through the stabilization of immunogenic RNA species (Huang T. et al., 2024).

The processes that are regulated by cytoplasmic readers include mRNA stability, decay, and translation, although these, too, can differ greatly across tumors (Schoenberg and Maquat, 2012). YTHDF1 promotes the translation of antigen-presentation machinery in colorectal cancer, which increases antitumor immunity but in gastric cancer (GC) serves as an immune checkpoint that enhances the immune response by boosting PD-L1 translation (Han D. et al., 2019). The transcriptional decays, YTHDF2, also exhibits bipolar behavior affecting a range of pro-survival transcripts in HCC, but stabilizing oncogenic EGFR signaling under hypoxia in lung cancer, and so highlights the capacity of microenvironmental stress to switch the functions of readers (Chen et al., 2023; Xu et al., 2025). IGF2BP family have tumor specific preferences in targeting: AML-IGF2BP1 stabilizes MYC (Elcheva et al., 2020), CRC-IGF2BP2 promotes glycolytic programs (Wang et al., 2019), whereas otomelanoma-IGF2BP3 induces invasion by stabilizing SOX10 (Liu J. et al., 2021). Since most ncRNAs are highly tissue/tumor-specific, their association in m6A readers constitutes discrete regulatory axes by type of cancer. As an example, YTHDF3 can stabilize lncRNA-based EMT programs to avoid cancer recurrence in breast cancer but stimulate the circRNA translation in liver metastasis models (Dattilo et al., 2023; Qin et al., 2021). Collectively, these findings highlight the necessity of considering the m6A specific-ncRNA networks at a compartment or tissue-specific level since their regulatory effects may be radically dissimilar despite being mediated by an identical reader protein.

## 3 Mechanisms of m6A in RNA stability, translation, and degradation

### 3.1 m6A and RNA stability

When m6A-modified RNA binds a particular “reader” protein, it determines the ultimate RNA destiny. YTHDF2 in the cytosol represents a fully documented m6A reading factor that leads to mRNA degradation. The CCR4-NOT deadenylase joins with decay complexes *via* the YTHDF2 Reader protein when it binds m6A-marked transcripts, which leads to poly(A) tail shortening and subsequent mRNA breakdown​ (Wang et al., 2023). In contrast, the IGF2BP1/2/3 family of readers locates m6A sites using their KH domains, which results in stabilizing their target mRNAs (Sun et al., 2022). The IGF2BPs collaborate with ELAVL1 (HuR) and other RNA-binding factors to maintain the stability of targeted transcripts. The reduction of cancer cell IGF2BP1-3 expression levels leads to faster degradation of oncogenic mRNAs MYC and FSCN1, which demonstrates their normal function in increasing the longevity of these expressed transcripts (Xie et al., 2021). YTHDC1 functions as a nuclear m6A reader that adds stabilizers like HuR to marked 3′UTRs through its reader activity (Wang S. et al., 2022).

**FIGURE 1:**
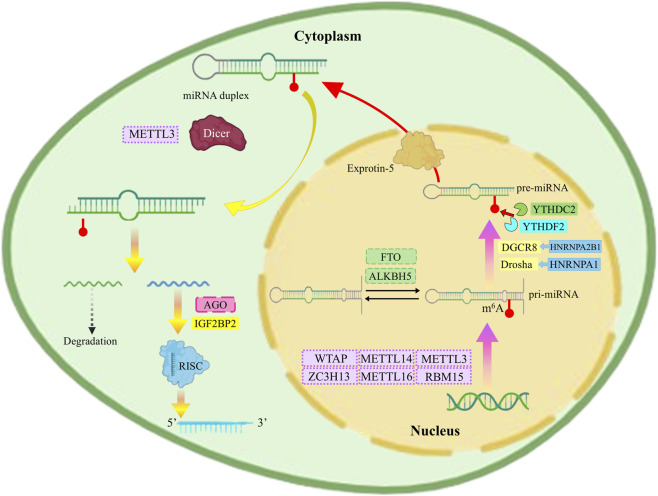
Types of m6A modification regulators in different cancer types, with red indicating oncogenic regulators, blue indicating anti-oncogenic regulators, and green indicating regulators with both oncogenic and anti-oncogenic effects.

m6A methylation determines RNA stability through opposing reader effects, which depend on both the cell environment and the reader proteins present in the system. The methylation status of m6A in many cancer cells transforms how oncogenes *versus* tumor suppressor mRNAs experience stability, highlighting the regulatory role of m6A modification in tumorigenesis (Figure 1). An excessive number of m6A modifications placed on tumor-suppressor mRNAs leads to YTHDF2 recognition, followed by fast degradation, which promotes tumor growth (Dixit et al., 2021). The abnormal stabilization of YTHDF2 in glioblastoma multiforme (GBM) through EGFR/SRC signaling allows the cells to decay tumor-suppressive m6A-bearing mRNAs such as LXRα and HIVEP2, which supports cancer cell growth (Fang et al., 2021). Readers tend to recognize m6A marks on select growth-promoting transcripts as they protect these transcripts from degradation. The GC protein IGF2BP3 links an m6A site located within HCC-derived growth factor (HDGF) mRNA, which protects the mRNA from decay processes, thus increasing HDGF protein levels to support tumor expansion and disease metastasis (Yu et al., 2021; Liu Z. et al., 2024). Similarly, the combination of IGF2BP3 with METTL3 leads to the m6A labeling of PD-L1 transcripts, which then become more stable, thus promoting increased expression levels of PD-L1 and blocking immune cell surveillance near tumors (Mu et al., 2024). Research has shown that m6A-mediated mRNA decay controls opposite cancer-related effects based on the specific scenario. YTHDF2 enhances EGFR mRNA decay in HCC cells through m6A modification of the 3′UTR, but deletion of YTHDF2 during hypoxic conditions leads to EGFR stabilization, which causes more HCC cell growth acceleration (Zhong et al., 2019). The impact of m6A modification on RNA stability in cancer depends on gene-specific expression patterns because the modification leads transcripts down instability pathways that activate oncogenic or suppress tumor development mechanisms.

Tumor cells show frequent changes in reader expression patterns where IGF2BP1/3 transcription factors resume fetal expression, but YTHDF2 can exhibit increased or decreased levels because these factors influence how m6A shapes cancer-associated RNA stability networks. Many studies have demonstrated how oncogenes like MYC, BCL2, and CDK6 would gain stability through reader interactions with m6A or how tumor suppressors like FOXO1 and PTEN would experience m6A-dependent decay in leukemia as well as breast cancer (BC) and liver cancer (Wan et al., 2022; Li D. et al., 2024). Overall, the m6A modifications function as robust regulatory elements because they both protect selected transcripts from decay while protecting other transcripts from destruction, thus influencing cancer cell proliferation patterns.

### 3.2 m6A and translation regulation

Catalyzed m6A marks serve translation-promoting proteins as their binding targets. The YTHDF1 protein recognizes methylated transcripts that lead to translation boosts through its recruitment or stabilization of translation initiation complex elements. YTHDF1 (together with YTHDF3) uses initiation factors including eIF3 and eIF4G/A to promote ribosome loading on mRNAs containing m6A marks (Li et al., 2017). In cytoplasmic capacity, METTL3 reads m6A signals to directly bind with translation initiation factors while forming an mRNA–ribosome complex through eIF3h, which enhances target protein synthesis without requiring methylation activity (Choe et al., 2018). The resulting mechanisms promote an overall elevation of protein production from m6A-marked messenger RNAs.

The overproduction of growth-related proteins in cancer cells results from m6A reader activities, which are also aberrant in translation control pathways. In liver and LC tissue, YTHDF1 specifically recognizes m6A-modifications on KEAP1 and NRF2 transcripts, which later leads to enhanced translation of both genes, thus affecting redox homeostasis in tumors (Wang et al., 2015). The translation elongation process receives support from pairwise YTHDF1 and YTHDF3 protein complex binding with translation apparatus components eIF4A/E to boost m6A-modified RNA-derived protein synthesis (Li et al., 2017; Meyer, 2019).

The m6A modifications located in 5′UTRs activate cap-independent translation, which cancer cells exploit under stressful conditions. Research results demonstrated that a single m6A modification in a 5′UTR region enables eukaryotic initiation factor 3 (eIF3) recruitment to an mRNA, thereby permitting the 43S pre-initiation complex formation independently of the m7G cap structure. After m6A is incorporated into an mRNA, it can generate an effect resembling an internal ribosome entry site. This process of translation requires no caps because cancer cells must continue protein production under hypoxic stress and mTOR inhibitory conditions (Meyer et al., 2015). The activity of METTL3 in placing m6A modifications in SNAI1 5′UTR allows HCC cells to synthesize the SNAIL protein during cap-dependent translation blockage through the binding of YTHDF1 together with elongation factor eEF2 for SNAI1 translation-mediated metastasis (Chang et al., 2019). The gut stromal tumor-related pathway shows METTL3 placing m6A marks on MRP1 5′UTR, which then attracts YTHDF1 to facilitate eEF1-based MRP1 translation increase (Shen et al., 2023). The resulting increase in MRP1 protein facilitates drug efflux and confers imatinib resistance. The examples show that m6A-dependent translation promotes tumor progression because it specifically increases the protein expression level of factors that encourage cancer advancement and dissemination, and treatment resistance.

Research indicates that m6A methylation dysfunction in cancer cells leads to elevated mRNA translation of ENO1 and SLC7A11 genes which drives glycolytic activity and ferroptosis blockage in LC cells (Kofuji et al., 2019), while PDK4 facilitates Warburg effect promotion in liver and cervical cancer (CC) tissues (Liu Q. et al., 2024), and NOTCH1 and MYC sustain leukemia stem-like cellular attributes (Huang et al., 2020). The readers of m6A carry out an amplification process of oncogenic signaling through improved translation efficiency for essential mRNAs. The link between m6A readers and translation represents a pivotal point in cancer cell biology, which allows malignancy-promoting protein synthesis modification.

### 3.3 m6A and RNA degradation

The major impact of m6A modification leads to degradation targeting. YTHDF2 functions as the main mediator during this process to recruit decay machinery by guiding m6A-modified mRNA to processing (P) bodies. The CCR4-NOT complex deadenylates mRNA tails, which are then degraded through exonucleolytic digestion or endonucleolytic cleavage (Park et al., 2023). The m6A decay regulation conducted by YTHDF2 initiates double parallel mechanisms that involve CCR4-NOT deadenylation in addition to HRSP12-MRP-mediated endoribonuclease digestion (Chen et al., 2023). The two decay pathways lead to the immediate elimination of transcripts.

The RNA degradation caused by m6A information results in two-fold mechanisms within cancer cells. Through m6A annotation, the cell can dispose of growth-suppressor and apoptosis-inducing transcripts, which enhances cancer cell survival, but also limits oncogenic RNA expression under specific circumstances (Duan et al., 2024). The overexpression of YTHDF2 in AML blasts results in the deterioration of differentiation and apoptotic mRNAs for leukemia stem cell (SCs) survival through m6A-dependent mechanisms (Paris et al., 2019). The AML pathogenic targets of YTHDF2 specifically include TNFR2 (TNFRSF1B) together with other mRNA transcripts that would naturally trigger cell death and SC loss (Paris et al., 2019). The functional clearance of these transcripts through YTHDF2 leads to the support of leukemogenesis. A cell can utilize m6A-marked oncogenic mRNA degradation by YTHDF2 to function as an antineoplastic mechanism, like how EGFR mRNA is destroyed in human HCC cells above. The cellular activity of m6A “erasers” (demethylases) determines whether the m6A methylation effects promote or suppress cell functions (Zhong et al., 2019). FTO works as an m6A mark eraser on oncogenic mRNAs, and FTO inhibition results in prolonged m6A modification that leads to their destruction. Research demonstrated that FTO inhibitor drugs used against leukemia and GBM enhance m6A marks on CEBPA and MYC oncogenes, which leads to YTHDF2-mediated mRNA destruction, resulting in tumor growth inhibition (You et al., 2022; Su et al., 2020). Research demonstrates that treating cells with m6A-mediated RNA degradation promotion results in desirable therapeutic outcomes.

Medical researchers study m6A-directed RNA decay dysregulation because it plays an essential role in tumor progression and supports experimental therapeutic approaches. Experimental studies indicate that tumor-suppressive genes regain stability and tumor growth becomes impaired when researchers delete or knock down YTHDF2, while overexpressing YTHDF2 accelerates tumor cell proliferation through inhibitory transcript clearance (Zhong et al., 2019; Li J. et al., 2020; Hou et al., 2021). When YTHDF2 expression is genetically removed in AML patients, the disease-specific leukemia SCs disappear without affecting normal hematopoietic SCs. AML cells need m6A-mediated transcript decay to survive, yet regular SCs remain unaffected because normal SCs possess the ability to withstand YTHDF2 loss (Murphy and Winters, 2023). The identified findings demonstrate that m6A-dependent degradation operates in specific contexts throughout cancer development.

The influence of YTHDF2 depends both on which transcripts receive marks and on how crucial those affected transcripts are to maintaining cell survival. The research has identified a relation between m6A RNA decay and the control of oncogenic signaling pathways. The methylation of β-catenin mRNA by IGF2BPs prevents YTHDF2 from causing its decay pathway, but such protection is lost when IGF2BP2 does not interact with β-catenin mRNA in hepatoblastoma and LC models, according to research established by a study (Qu et al., 2024). The constant struggle between these two factors determines how cancer originates and spreads through the body. Researchers are developing drug targets from degradation components in the m6A pathway. Decay rates of specific m6A-marked RNAs might be adjustable with downstream nucleases such as RNase P/MRP and XRN exonucleases. Preclinical cancer research indicates modified ASOs hold the potential to create m6A marks at select sites or attract YTHDF2 toward cancer-causing messenger RNAs to achieve targeted degradation of these transcripts (Jaiswal et al., 2024). Overall, the controlled breakdown of RNAs marked with m6A functions as an essential regulatory system that cells use to adjust gene expression, and tumor growth benefits from thwarting this mechanism. Scientists actively explore m6A-regulated decay events to identify those that promote or suppress cancer development because they lead to significant therapeutic opportunities.

Recent works on metabolic labelling show that m6A dimensions the lifetime of ncRNAs in a highly dynamic fashion by altering the half-lives of RNAs using reader competition. YTHDF2 increases the kinetics of degradation, but IGF2BP proteins increase the ncRNA persistence, establishing kinetic tug-of-war systems, which control cancer plasticity and therapy response. This change in turnovers takes place quickly when conditions like hypoxia and oncogenic activation of pathways change, allowing cancer cells to reorganize ncRNA landscapes in response (Wiener and Schwartz, 2021; Sun et al., 2024).

m6A does not only regulate ncRNA activity by recruiting readers, but also by regulating local renal RNA transcripts to change access to RNA-binding motifs and change ribonucleoprotein (RNP) assembly. This is referred to as the m6A-switch, which happens when methylation destabilizes A-U base pairing or short stems, and opens single-stranded, previously occupied motifs to protein docking (Jin and Fan, 2024). The initial model of MALAT1 has demonstrated that hnRNPC/HNRNPG binding occurs when the M6A of adjacent U-tracts arises because of the solvent accessibility of the original U-tracts by solvents (Liu et al., 2017). m6A in stem loops in pri-miRNAs is necessary to stabilize a structurally permissible conformation facilitating DGCR8 recognition and Microprocessor loading to promote miRNA maturation. This structural facilitation is the reason that processing oncogenic pri-miRNA is impaired by the loss of METTL3/METTL14 in a variety of tumors (Huang et al., 2020).

Structural switching controls assembling of lncRNA-protein scaffolds in lncRNAs. Indicatively, m6A-regulated dispersal of local helices in MALAT1 or XIST uncovers RBP docking sites (hnRNPC, RBMX, YTHDC1) that allow valuable formation of either export, splicing, or deamidification complexes depending on context. In certain instances, the re-modeled states enable the reader proteins to act on lncRNAs and induce selective degradation, but other forms of RBP engagement either stabilize them or cause them to become oncogenic (Jones et al., 2022; Zhao et al., 2021). Single or clustered m6A residues in circRNAs may both be structural and functional landing pads. M6A may augment accessibility of reader-binding motifs by destabilizing closed intramolecular folds to promote either cap-independent translation due to recruitment of YTHDF3/eIF4G2 or IGF2BP docking due to circRNA stabilization. Therefore, the eventual outcome of an m6A-specifically modified circRNA, translation, export, or decay highly depends on the structural accessibility catalyzed by methylation and the repertoire of readers in that tumor lineage (Covelo-Molares et al., 2018). Taken together, these results show that m6A is a structure-first regulator, and ncRNA folding alters before and determines which protein complexes form, providing a structural simplification in m6A crosstalk with ncRNAs in oncogenesis.

## 4 Mutual regulation between m6A modifications and ncRNAs

### 4.1 Mutual regulation of miRNAs and m6A modifications

miRNAs are single-stranded ncRNA molecules that are involved in post-transcriptional gene regulation (Li W. et al., 2022). They regulate genes by binding to the 3′ and 5′ untranslated regions (UTRs) and coding region of target mRNAs, resulting in translational inhibition or degradation of the mRNA (Zhou et al., 2024). They possess the ability to bind to gene promoter regions to activate transcription in some miRNAs, suggesting a more general regulatory function (Broughton et al., 2016). There is even growing evidence that m6A-mediated ncRNA regulation is not dropped into chromatin homeostasis. The recruitment of METTL3 to H3K36me3-marked transcriptional units, however, allows co-transcriptional methylation of pri-miRNAs and lncRNAs, and YTHDC1 mediates the connection between the m6A-RNA recognition and the H3K9me2 demethylation and demethylations into H3K9me2 by isoquinolates and transcriptional activity through the non-catalytic binding of KDM3B (Liu XM. et al., 2022; Xu Z. et al., 2022). Therefore, ncRNA-m6A cues are built into the spatial position of histone modification modes, and together form oncogenic transcriptional programs.

miRNA biogenesis starts with the transcription of pri-miRNA by RNA polymerase II to yield capped and polyadenylated transcripts (Lee et al., 2004). pri-miRNAs are cleaved in the nucleus by the Drosha-DGCR8 complex into ∼65-nucleotide hairpin precursor miRNAs (pre-miRNAs) (Han et al., 2004). Pre-miRNAs are exported to the cytoplasm by Exportin-5 in a Ran-GTP-dependent manner (Yi et al., 2003). Within the cytoplasm, Dicer processes the pre-miRNAs to ∼22-nucleotide duplexes (Saito et al., 2005), the guide strand of which is loaded into the RNA-induced silencing complex (RISC) with Argonaute proteins, and the passenger strand is typically degraded (Borges and Martienssen, 2015). Mature miRNAs in the RISC complex mainly interact with the 3′UTRs of target mRNAs to play a role in gene silencing (Valinezhad Orang et al., 2014).

**FIGURE 2:**
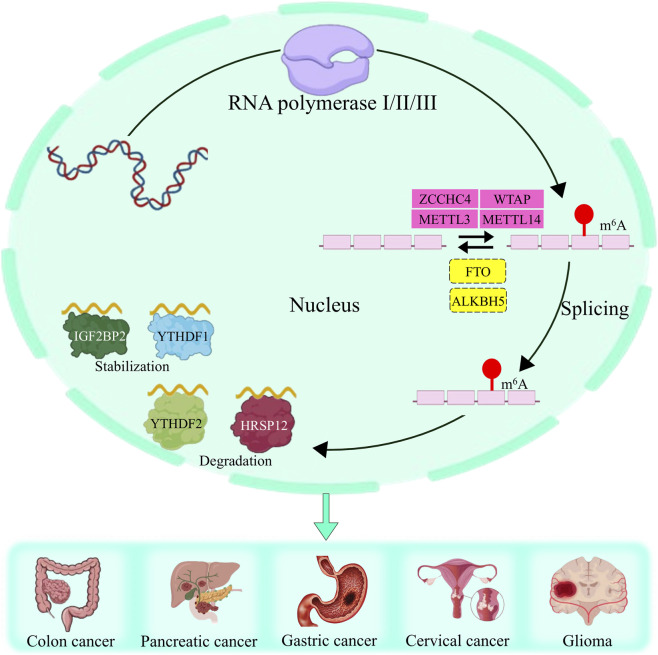
m6A alterations on miRNAs are introduced by m6A writers and eliminated by m6A erasers. Concurrently, m6A-modified miRNAs are identified and bound by m6A readers, which are essential in controlling miRNA maturity. Moreover, METTL3 directly associates with Dicer, HNRNPA2B1 engages DGCR8, HNRNPA1 amplifies Drosha movement, and IGF2BP2 attaches to AGO proteins, thereby influencing the complex control of miRNA development.

Given the complexity of miRNA biogenesis, multiple regulatory layers are required to maintain its accuracy and adaptability to cellular signals. One of these includes m6A, the most abundant internal modification in eukaryotic RNAs, which controls splicing, export, stability, and translation of the RNA (Dai et al., 2018). Emerging evidence uncovers context-dependent, bidirectional regulation between m6A regulators and particular miRNAs, forming complex feedback loops that control cancer cell fate. Such interplay extends beyond conventional gene regulation, remolding pri-miRNA maturation, methylation dynamics across the transcriptome, and oncogenic signal transduction cascades. Dysregulation of these regulatory interactions is the basis for some important oncogenic properties, such as chemoresistance (Ding et al., 2024), metastatic dissemination (Qian et al., 2023), and immune evasion in certain cancers. Table 1 summarizes representative examples of these mutual regulatory axes, detailing how m6A modifications and miRNAs coordinately modulate each other’s expression and function in cancer. Figure 2 illustrates that m6A alterations on miRNAs are introduced by m6A writers and eliminated by m6A erasers.

**TABLE 1:** Mutual regulation between miRNAs and m6A modifications in cancer.

Cancer type	miRNA(s)	m6A regulator(s)	m6A modification role	Regulatory mechanism	Functional outcome	References
Bladder cancer	miR-221/222	METTL3, DGCR8	Methylation of pri-miR-221/222	METTL3 promotes m6A modification and maturation of pri-miR-221/222	Promotes proliferation by suppressing PTEN	Han et al. (2019b)
Ovarian cancer	miR-30c-5p	HNRNPA2B1	Reduction of m6A levels	miR-30c-5p suppresses HNRNPA2B1, decreasing global m6A modification	Suppresses proliferation, migration, and invasion	Wu et al. (2023b)
Prostate cancer	miR-493-3p	YTHDF2	Increase in m6A levels	miR-493-3p targets YTHDF2, leading to higher m6A and reduced malignancy	Inhibits proliferation and migration	Li et al. (2018)
Acute Myeloid Leukemia	miR-493-5p	METTL3	Reduction of m6A on MYC mRNA	miR-493-5p suppresses METTL3, reducing m6A levels on MYC	Decreases chemoresistance and tumor progression	Wang et al. (2022c)
Colorectal cancer	miR-17-5p	METTL14, YTHDC2	Loss of m6A on pri-miR-17	METTL14 suppresses miR-17-5p maturation while miR-17-5p promotes chemoresistance	Enhances 5-FU resistance	Sun et al. (2023)
Hepatoblastoma	miR-186	METTL3	Suppression of m6A through METTL3 downregulation	miR-186 targets METTL3, reducing its expression and influencing Wnt/β-catenin pathway	Inhibits proliferation and invasion	Cui et al. (2020)
Choriocarcinoma	miR-21-5p	METTL3	Methylation and maturation of pri-miR-21-5p	METTL3 promotes miR-21-5p maturation; miR-21-5p suppresses HIF1AN	Promotes tumor growth and EMT	Ye et al. (2022c)
Non-Small Cell Lung Cancer	miR-21-5p	METTL3	Methylation of pri-miR-21-5p	METTL3 facilitates miR-21-5p maturation, which targets FDX1	Promotes proliferation and metastasis	Qian et al. (2023)
Lung adenocarcinoma	miR-138-5p	FTO, DGCR8	Demethylation of pri-miR-138-5p	FTO reduces m6A on pri-miR-138-5p, impairing DGCR8 recognition	Promotes gefitinib resistance	Ding et al. (2024)
Clear Cell Renal Cell Carcinoma	miR-155	FTO	Increase in global m6A levels	miR-155 suppresses FTO translation, increasing overall m6A	Enhances proliferation, inhibits apoptosis	Yang et al. (2022b)
Glioblastoma	miR-145	FTO, AGO1, ILF3	Demethylation of mRNAs *via* motif-specific targeting	miR-145 recruits a complex including FTO to RRACH motifs, removing m6A	Promotes translation of tumor suppressors	Zepecki et al. (2021)
Glioblastoma	miR-10a	FTO, HNRNPA2B1, DGCR8	Methylation of pri-miR-10a	Suppression of FTO enhances m6A on pri-miR-10a, promoting its maturation	Promotes tumor growth and progression	Zhang et al. (2022b)
Osteosarcoma	miR-451a	YTHDC1	m6A-mediated mRNA stabilization	miR-451a targets YTHDC1, altering stabilization of PDPK1 mRNA	Inhibits tumor progression *via* AKT/mTOR signaling	Cao et al. (2022)

### 4.2 Mutual regulation of lncRNAs and m6A modifications

LncRNAs are RNA transcripts with more than 200 nucleotides. The length criterion is most often utilized to separate them from the shorter RNAs, like 5S ribosomal RNAs, transfer RNAs, miRNAs, and PIWI-interacting RNAs during the process of RNA purification (Mercer et al., 2009). lncRNAs may also form intricate secondary and tertiary structures like hairpins, loops, and stems. These complexes allow for interaction with proteins, RNA, and DNA, for many biological processes (Cruz and Westhof, 2009). lncRNAs share some features with messenger RNAs. They are processed by RNA polymerase II, possess a 5′ cap and 3′ polyadenylated tail, and frequently undergo canonical splicing (Rutenberg-Schoenberg et al., 2016). These structural features enable lncRNAs to function as molecular scaffolds, decoys, or guides. They can recruit chromatin remodelers or transcription factors to targeted genomic positions and regulate gene expression without coding for proteins. m6A has been recognized as a prevalent modification in lncRNAs in recent research. The modification can reform RNA structure and control the function of lncRNAs in several regulatory processes (Gilbert et al., 2016).

**FIGURE 3:**
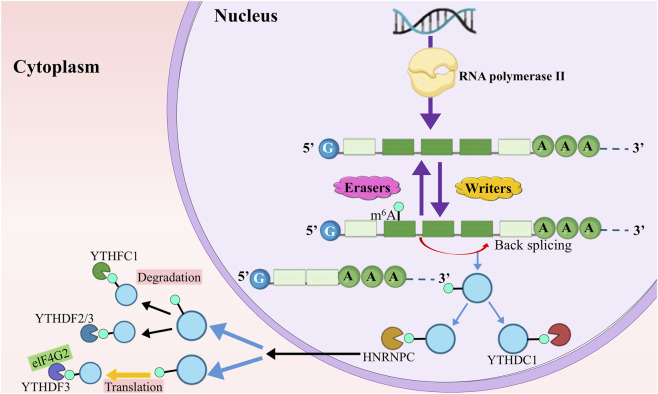
m6A writers facilitate the incorporation of m6A alterations into lncRNAs, whereas m6A erasers are responsible for their elimination. Concurrently, m6A readers attach to the m6A domains on lncRNAs, thereby modulating their ability to survive and break down. The complex system of m6A alterations is essential in the advancement of several malignancies.

m6A modifications also directly affect lncRNA and RNA-binding protein (RBP) interaction, a process known as the “m6A switch”. For example, m6A methylation changed the duplex structure of MALAT1 and controlled binding to HNRNPC and HNRNPG, demonstrating how m6A controls protein-lncRNA interactions (Liu et al., 2017). In addition, m6A reader YTHDC1 was found to enable the export of m6A-modified lncRNAs from the nucleus by its interaction with the TREX complex, enabling appropriate location and function (Qiao et al., 2023). In LUAD, wherein oncogenic lncRNA LCAT3 was stabilized through METTL3-directed m6A modification. This resulted in enhanced transcript accumulation, enhanced cell proliferation, migration, and tumor growth. LCAT3 subsequently recruited FUBP1 to activate MYC transcription, thereby creating a feedback loop that enhanced tumorigenicity (Wang et al., 2021). Moreover, in HNSCC, the lnc-H2AFV-1 was found overexpressed and elevated global m6A levels by enhancing METTL3/14 and repressing eraser FTO. It also enhanced IFT80 expression by enriching m6A, creating another feedback loop between the lncRNA and the m6A machinery (Chen X. et al., 2022). Similarly, in endometrioid endometrial carcinoma (EEC), lncRNA FENDRR was downregulated while its m6A levels were elevated. YTHDF2 recognized the methylated FENDRR and promoted its degradation, diminishing its tumor-suppressive effects, including inhibition of SOX4 and cell proliferation (Shen et al., 2021). In PCa, lncRNA PVT1 was found to be highly expressed and epigenetically stabilized by METTL3-mediated m6A modification. PVT1 promoted cell proliferation, invasiveness, and migration by sponging miR-27b-3p, which released the repression of BLM, a downstream target in cancer progression (Chen et al., 2022c). Table 2 summarizes cancer-specific m6A–lncRNA axes, highlighting their regulators, modification roles, mechanisms, and effects on tumor biology. Figure 3 illustrates that m6A writers facilitate the incorporation of m6A alterations into lncRNAs, whereas m6A erasers are responsible for their elimination.

**TABLE 2:** Mutual regulation between lncRNAs and m6A modifications in various cancers.

Cancer type	lncRNA(s)	m6A regulator(s)	m6A modification role	Regulatory mechanism	Functional outcome	References
Bladder Cancer	KCNQ1OT1, SNHG16, AC097359.2, AC097641.2, EHMT2-AS1, AC006160.1, MED28-DT, AC116914.2, MYOSLID, AC104564.3, BDNF-AS	METTL3, RBM15	Stabilization	Upregulation of METTL3 and RBM15 enhances m6A levels and stabilizes prognostic lncRNAs	Promotes tumor cell proliferation, migration, invasion; poor prognosis	Huang et al. (2024b)
Gastrointestinal Cancer	FAM83H-AS1	METTL3, IGF2BP2, IGF2BP3, PTBP1	Stabilization	METTL3-mediated m6A enhances FAM83H-AS1 stability; interacts with PTBP1 to regulate splicing	Enhances metastasis and therapy resistance	Luo et al. (2024)
Gastric Cancer	TP53TG1	ALKBH5	Degradation	ALKBH5 reduces m6A levels on TP53TG1, decreasing its stability	Suppresses PI3K/AKT signaling; induces apoptosis	Fang et al. (2022)
Prostate Cancer	MALAT1	METTL3	Stabilization	METTL3 methylates MALAT1 and increases transcript stability	Activates PI3K/AKT pathway; promotes proliferation	Mao et al. (2022)
Nasopharyngeal Carcinoma	DIAPH1-AS1	WTAP, IGF2BP2	Stabilization	WTAP adds m6A marks; IGF2BP2 binds and stabilizes DIAPH1-AS1	Promotes metastasis *via* MTDH-LASP1 complex	Li et al. (2022d)
Colorectal Cancer	POU6F2-AS1	METTL3, YBX1	Stabilization	m6A-modified POU6F2-AS1 recruits YBX1 to the FASN promoter to drive transcription	Promotes lipogenesis and tumor growth	Jiang et al. (2024b)
Prostate Cancer (Metastatic)	PCAT6	METTL3, IGF2BP2	Stabilization	METTL3 modifies PCAT6; IGF2BP2 enhances IGF1R mRNA stability	Promotes bone metastasis	Lang et al. (2021)
Hepatocellular Carcinoma	AC115619	WTAP	Global m6A reduction	AC115619-22aa peptide binds WTAP and disrupts the methyltransferase complex	Inhibits tumor growth and gene methylation	Zhang et al. (2023a)
Breast Cancer	UCA1	METTL14 (silenced by methylation)	Silencing	UCA1 epigenetically represses METTL14; reduces m6A on miR-375 leading to miR-375 degradation	Increases SOX12 expression; promotes proliferation	Zhao et al. (2022)
Non-Small Cell Lung Cancer	MEG3	HNRNPA2B1	Degradation	m6A-modified MEG3 destabilized by HNRNPA2B1; affects PTEN through miR-21-5p	Enhances metastasis and proliferation	Li et al. (2023b)
Non-Small Cell Lung Cancer	DGUOK-AS1	METTL3, IGF2BP2	Stabilization	m6A enhances TRPM7 mRNA stability *via* DGUOK-AS1–IGF2BP2 complex	Promotes tumor growth and metastasis	Feng et al. (2023)
Non-Small Cell Lung Cancer	CALML3-AS1	ALKBH5, YTHDC2	Stabilization and Destabilization	ALKBH5 stabilizes CALML3-AS1; YTHDC2 binds and destabilizes it	Suppresses BTNL9; enhances metastasis	Zhang et al. (2023b)
Colorectal Cancer	ABHD11-AS1	METTL3, IGF2BP2	Stabilization	ABHD11-AS1 stabilized by m6A; enhances IGF2BP2/TRIM21 interaction; indirectly stabilizes FOXM1	Inhibits ferroptosis; promotes migration and proliferation	Bian et al. (2024)
Gastric Cancer	lnc-PLCB1	METTL14	Destabilization	H. pylori–induced METTL14 methylates and downregulates lnc-PLCB1	Promotes CCND1 and Slug expression; drives progression	Chang et al. (2024)
Colorectal Cancer	MIR100HG	hnRNPA2B1	Stabilization	m6A-modified TCF7L2 mRNA stabilized *via* MIR100HG–hnRNPA2B1 interaction	Induces EMT, metastasis, and cetuximab resistance	Liu et al. (2022b)
Gastric Cancer	THAP7-AS1	METTL3, IGF2BP1	Stabilization	m6A increases THAP7-AS1 stability; facilitates CUL4B nuclear entry and miRNA repression	Activates PI3K/AKT signaling; promotes metastasis	Liu et al. (2022c)
Gastric Cancer	CBSLR	YTHDF2	Degradation	m6A-bound CBS mRNA degraded *via* CBSLR–YTHDF2 interaction under hypoxia	Inhibits ferroptosis; increases chemoresistance	Yang et al. (2022c)
Hepatocellular Carcinoma	MIR155HG	METTL14, ELAVL1 (HuR)	Stabilization	METTL14 methylates MIR155HG; ELAVL1 binds and stabilizes it	Activates PD-L1 *via* miR-223 sponging and STAT1 signaling	Peng et al. (2022)

### 4.3 Mutual regulation of circRNAs and m6A modifications

CircRNAs are a novel class of RNA molecules formed by back-splicing of pre-mRNA lacking 5′ cap and 3′ poly (A) tail (Pisignano et al., 2023). circRNA is more stable because, being circular in structure, it is not susceptible to degradation by RNA nucleases (Zhang et al., 2016). Though most circRNAs are abundantly expressed in the cytoplasm, few are found within the nucleus (Kristensen et al., 2019). They perform a wide range of regulatory functions, such as acting as ceRNAs that bind to miRNAs and thus influence the level of the downstream targeted mRNAs. They also function as transcription modulators and protein scaffolds to enable their regulatory mechanisms (Zhou W-Y. et al., 2020). Notably, in some cases, circRNAs might even have coding capacity (!!! INVALID CITATIONa). Recent dedicated reviews have systematically profiled circRNA expression across multiple tumor types and outlined their emerging diagnostic and prognostic value in oncology (Khan et al., 2022a). In parallel, a comprehensive analysis of circRNA-encoded chimeric peptides/proteins has highlighted how many of these translation products, often initiated *via* IRES or m^6^A-dependent mechanisms, directly modulate proliferation, invasion and therapeutic response in cancer (Khan et al., 2022b). These studies support our emphasis on m^6^A-regulated circRNA biology and underscore the translational potential of circRNA signatures and circRNA-encoded peptides as biomarkers and therapeutic targets.

**FIGURE 4:**
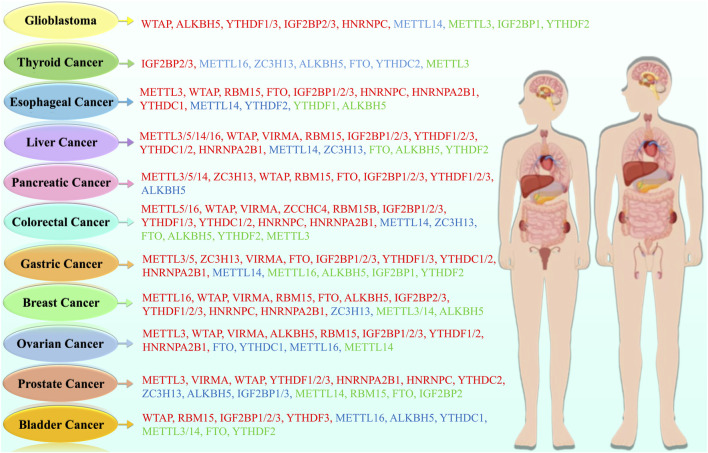
m6A writers facilitate the incorporation of m6A modifications into circRNAs, whereas m6A erasers are tasked with the removal of these changes. Moreover, m6A readers govern several facets of circRNA biology, involving their synthesis, exportation, translation, and destruction, *via* unique attachment to m6A spots on circRNAs.

Remarkably, m6A alterations and circRNAs have regulatory bidirectional interactions with one another that impact key processes such as circRNA biogenesis, translation, and degradation (Figure 4):

#### 4.3.1 Generation of circRNAs

m6A methylation has also been engaged in circRNA biogenesis in various cancers. In PDAC and CRC, circRNA biogenesis is controlled by METTL3, which binds flanking sequences to promote tumor initiation (Chen C. et al., 2021; Zeng et al., 2023). In NSCLC, METTL3 facilitated m6A modification of circIGF2BP3, promoting its reverse splicing and circularization in a YTHDC1-dependent manner to enable immune escape by inducing PD-L1 deubiquitination (Liu et al., 2021b). In PC, WTAP regulated m6A modification, which affected pre-mRNA splicing and circDDIT4 generation, promoting tumor growth (Kong et al., 2023). In HCC, YTHDC1 was found to stimulate circRNAs to reverse splicing and biogenesis, which is responsible for hepatocarcinogenesis (Rao et al., 2021; Wei et al., 2023). Moreover, in chronic lymphocytic leukemia (CLL), YTHDC1 and RBMX controlled CLL growth through interaction with circTET2 flanking sequences, collectively facilitating splicing and recycling of exon 3 to produce circTET2 (Wu Z. et al., 2023).

#### 4.3.2 Translation of circRNAs

Although circRNAs in general are classified as ncRNAs, several cytoplasmic circRNAs have translation potentials. Two major mechanisms of translation have been noticed: one is internal ribosome entry site (IRES)-mediated and the second is m6A modification-mediated (Wen et al., 2022; Diallo et al., 2019). The m6A-mediated mechanism relies on the m6A reader YTHDF3 and the eIF4G2 translation initiation factor for translation recruitment (Diallo et al., 2019). In liver metastasis-associated CRC, circYAP has been shown to recruit the eIF4G2 complex through YTHDF3 to enable efficient translation (!!! INVALID CITATIONb). Moreover, Dattilo et al. have observed that m6A modification can induce cap-independent translation of circ-ZNF609, which is mediated by YTHDF3 and eIF4G2 and modulates its metabolic fate (Di Timoteo et al., 2020).

#### 4.3.3 Cytoplasmic export of circRNAs

circRNAs are predominantly cytoplasmic-localized, underlining the significance of effective nuclear exportation processes. METTL3 was shown to facilitate cytoplasmic exportation of m6A-modified circTEAD1 and thus regulate chordoma tumorigenesis (Li H. et al., 2024). In OC, YTHDC1 promoted the cytoplasmic translocation of hsa-circ-0061179 by direct binding with its m6A modification site and subsequent regulation of DNA damage and apoptosis (Zhang et al., 2024a). Furthermore, YTHDC1 also interacted with hsa-circ-0058493 in HCC to facilitate its cytoplasmic export, accelerating disease severity (Wu et al., 2021). Furthermore, in Wilms’ tumor, HNRNPC facilitated the release of m6A-modified circMARK2, stabilizing LIN28B mRNA and playing a role in tumor growth (Shu et al., 2024).

#### 4.3.4 Degradation of circRNAs

Although circRNAs are highly structurally stable, recent research has identified certain specific cancer degradation mechanisms. Among these are the endonuclease DIS3, which was found to specifically cleave circRNAs in the cytoplasm irrespective of the RNA exosome complex. DIS3 knockdown induced the upregulation of more than 60% of circRNAs, especially U-rich motif-enriched circRNAs, indicating a sequence-specific, conserved circRNA decay process in cancer cells (Tao et al., 2025). Besides endonucleolytic cleavage, m6A modification is also found to be a key epitranscriptomic regulator of circRNA stability. In CRC, circ_0003215 was markedly downregulated by m6A-induced degradation through YTHDF2 and was associated with increased tumor size and more advanced clinical stage. Functionally, circ_0003215 was a tumor suppressor that functioned to sponge miR-663b to upregulate DLG4 and suppress the pentose phosphate pathway (PPP) by ubiquitination of G6PD, connecting circRNA degradation to metabolic regulation (Chen et al., 2022d). Similarly, circAFF2 was shown to undergo m6A-dependent degradation mediated by YTHDF2. ALKBH5, a demethylase for m6A, repressed its radiosensitivity and restored stability by blocking Cullin neddylation, indicating the dynamic turnover of circRNA for therapeutic response (Shao et al., 2023). In NSCLC, circ_SFMBT2 (circ_0017628) was methylated and degraded after binding with YTHDF2, and silencing YTHDF2 restored its expression. Upregulated circ_SFMBT2 inhibited NSCLC cell growth and EMT by activating the Hippo/YAP pathway, and its tumor-suppressive effect was positively correlated with LATS2 expression (Xu J. et al., 2022).

circRNAs are also involved in the regulation of expression of m6A regulatory proteins and thus in disease development. For example, in HCC, circRERE downregulation increased m6A levels of GBX2 and activated its expression by enhancing the METC ZC3H13 (Lin Y-h et al., 2022). Likewise, in AML, circ-0001187 served as a sponge to miR-499a-5p, triggering upregulation of the E3 ubiquitin ligase RNF113A, resulting in proteasomal degradation of METTL3 and inhibition of leukemogenesis (Yang et al., 2023). CircRNAs significantly influence the targeting and regulation of m6A erasers. For example, in EOC, circRAB11FIP1 facilitated tumor growth and invasion by enhancing autophagic flux. It controlled ATG7 and ATG14 through sponging miR-129 and direct upregulation of the m6A eraser FTO, thus controlling m6A-dependent expression of ATG5 and ATG7 (Zhang Z. et al., 2021). Circ-0005615 increases m6A levels of MAP3K4 by reducing ALKBH5, resulting in decreased MAP3K4 and accelerated growth of multiple myeloma (Zhu et al., 2024).

circRNAs also regulate m6A readers in various cancers. For instance, in HCC, circMAP2K4 sponges hsa-miR-139-5p to overexpress the m6A reader YTHDF1 to promote cancer cell proliferation (Chi et al., 2021). Similarly, in breast cancer, circBACH2 (hsa-circ-0001625) increased HNRNPC by sponging hsa-miR-944, enhancing the MAPK signaling pathway, and driving tumor growth (Lv et al., 2021). Furthermore, circRNAs also directly target the IGF2BP of m6A readers. In NSCLC, circNDUFB2 was found to decrease the stability of IGF2BP *via* a ubiquitin proteasome system, thus repressing cancer proliferation (Li B. et al., 2021). In bladder cancer, circPTPRA interacted with KH3 and KH4 sites of IGF2BP1 and inhibited its interaction with downstream m6A-modified mRNAs and inhibited cancer development (Xie et al., 2021).

### 4.4 Mutual regulation of siRNAs and m6A modifications

The mutual regulation between m6A modification and siRNA-induced silencing is important for cancer development and suppression, which provides new targets for therapy. Overexpression of circMDK through excessive m6A methylation of hsa_circ_0095868 (circMDK) was identified to promote the proliferation and metastasis of HCC. Poly (β-amino esters) (PAEs) were used as carriers of circMDK-targeting siRNA to effectively inhibit circMDK expression and tumor growth in various HCC models (Du et al., 2022). Furthermore, METTL3-mediated m6A modification was reported to stabilize LINC00958, a lipogenesis-associated lncRNA, facilitating HDGF-mediated tumor growth. A PLGA nanoplatform containing si-LINC00958 exhibited efficient tumor targeting and controlled release, showing remarkable inhibition of tumor growth (Zuo et al., 2020).

The m6A and siRNAs interaction has also been implicated in other cancers, such as LUAD, TNBC, and GC. In LUAD, METTL3-mediated m6A modification of lncRNA AC098934 increased its stability and thus promoted cell proliferation, migration, and invasion. Knockdown of AC098934 with siRNAs (si-AC098934-1 and si-AC098934-2) strongly inhibited the proliferation and metastasis of LUAD cells *in vitro* and *in vivo* (Huang et al., 2022). In TNBC, METTL3 was observed to suppress COL3A1 expression through m6A methylation and thus inhibit cancer cell invasion and metastasis. Conversely, knockdown with METTL3 siRNA decreased m6A in COL3A1 mRNA, increasing its stability and inducing metastasis (Shi et al., 2020). In addition, an advanced therapeutic strategy in GC involved the use of engineered small extracellular vesicles (sEVs) with high expression of CD47 and cyclic arginine–glycine–aspartic (c (RGDyC)) modification to deliver siRNA against YTHDF1, an m6A reader. Silencing YTHDF1 interfered with m6A-dependent stabilization of Frizzled7 mRNA, inhibiting the Wnt/β-catenin pathway and inducing an immune response through increased major histocompatibility complex class I (MHC-I) expression, thus enhancing tumor cell phagocytosis (You et al., 2023).

### 4.5 Mutual regulation of PIWI-Interacting RNAs (piRNAs) and m6A modifications

piRNAs are emerging as key regulators of cancer progression, particularly in their association with m6A RNA modifications. In CC, piRNA-14633 overexpression was found in tumor tissues and cell lines, which triggered malignant traits such as cell viability, growth, migration, and invasion. Mechanistically, piRNA-14633 enhanced m6A RNA methylation through stabilizing METTL14, a pivotal m6A METC. Dual-luciferase reporter assays validated that piRNA-14633 targeted METTL14 directly and, after knocking down METTL14, reversed oncogenic effects. *In vivo* assays also confirmed the tumor-promoting activity of piRNA-14633 (Xie et al., 2022). Moreover, piRNA-17458 was also identified to promote the m6A level through the stabilization of WTAP, an important component of the m6A METC. Dual-luciferase reporter assays further demonstrated that piRNA-17458 specifically targeted WTAP, and piRNA-17458 knockdown suppressed oncogenic activities, highlighting the potential to target piRNA-17458 in CC (Liu et al., 2023). In LC, piR-27222 has been shown to induce resistance against PANoptosis by stabilizing WTAP and increasing m6A modification of Casp8 transcripts. Such stabilization lowered the degradation of the transcript, thus further substantiating the oncogenic function of piRNA-27222 in the induction of LC development (Ma et al., 2024).

In BC, piRNA-31106 was significantly upregulated and promoted tumorigenesis through increased m6A methylation and overexpression of METTL3. The interaction was validated using RNA immunoprecipitation assays, demonstrating that piRNA-31106 interacted with METTL3 directly, and METTL3 knockdown suppressed the oncogenic activities (Huang et al., 2023). Additionally, in TNBC, piR-31115 promoted angiogenesis by m6A modification of YAP1, a key transcriptional regulator of tumor vasculature. By stabilizing YAP1, piR-31115 promoted endothelial cell proliferation and migration (Du et al., 2025). In OC, piR-26441 was found to be an important mediator of mitochondrial oxidative phosphorylation and tumor growth. PiR-26441 downregulation suppressed tumor growth, while overexpression enhanced m6A levels and induced TSFM mRNA degradation, resulting in mitochondrial dysfunction, increased ROS, and apoptosis. The effect was YTHDC1-dependent, linking piRNAs to m6A modifications and mitochondrial metabolism in OC (Yuan J. et al., 2025).

### 4.6 Mutual regulation of small nuclear RNAs (snRNAs) and m6A modifications

Although the regulatory functions of snRNAs and m6A modifications in cancer are increasingly evident, much is yet to be explored about their complex interactions and their roles as treatments. Recent evidence has underscored their important roles in cancer development and treatment outcomes. In clear cell renal cell carcinoma (ccRCC), for example, lncRNA DRAIC was found to stabilize hnRNPA2B1 through repression of its ubiquitination and proteasomal degradation, enhancing m6A-dependent IGF1R mRNA destabilization and suppressing tumor growth (Wen et al., 2024). Similarly, in HCC, m6A-modified snRNAs were found to be independent predictors of prognosis, and a risk model revealed that high-risk patients had worse overall survival and higher vulnerability to immunotherapy, as indicated by higher TIDE scores (Zhang C. et al., 2022). Furthermore, in LUAD, spliceosome-associated snRNA SNRPA was found to play a crucial role in chemotherapy resistance through the control of alternative splicing of ERCC1 exon 8. This was further controlled by m6A readers such as IGF2BP1 and RNA stabilizers such as ELAVL1 (Fan et al., 2024).

### 4.7 Mutual regulation of tRNA-derived small RNAs (tsRNAs) and m6A modifications

The regulatory functions of tsRNAs and their mechanism of interaction with m6A modifications remain a highly ongoing research area that requires further investigation. Research on acute pancreatitis (AP) found that tRF36 was significantly upregulated in AP patients’ serum and controlled ferroptosis by binding IGF2BP3 to p53 mRNA’s m6A modification site, increasing its stability and causing pancreatic cell ferroptosis, facilitating the development of AP (Fan et al., 2023). Another study also unveiled the regulatory function of tRF-16 in LC development, where its downregulation was correlated with increased migration, invasion, and radioresistance. Mechanistically, TRF-16 interfered with the IGF2BP1-CPT1A interaction that suppresses fatty acid metabolism and cancer cell proliferation *in vivo* (Ye J. et al., 2024). However, these findings highlight that the pathways through which tsRNAs modulate the function of m6A modifications are still unknown, and more research in this direction can uncover new targets for cancer therapy and other related diseases.

### 4.8 Contradictory roles of m6A regulators in tumor promotion and suppression

Though m6A regulators have frequently been characterized as either uniformly oncogenic or tumour-suppressive, there has been mounting evidence to suggest that their biological actions are highly context-dependent, and often have a conflicting outcome in various cancer types. Such inconsistencies are due to the variability of cellular state, metabolic environment, ncRNA abundance, which is caused by the preponderance of certain reader proteins. METTL3 has been reported to be oncogenic, which facilitates the progression of tumor through the support of MYC, BCL2, SOX2, and lncRNA in AML, LUAD, and breast cancer (Zeng et al., 2020). In CRC, however, METTL3 has a tumor-suppressive phenotype through the maturation of the anti-metastatic miR-1246 and stabilizing SOCS2 mRNA and thus suppressing JAK/STAT signaling (Li et al., 2019). On the other hand, METTL14 is a generally tumor-suppressive gene that facilitates hepatocarcinogenesis by increasing the m6A-mediated processing of oncogenic pri-miRNAs in hepatic progenitor cells (Fan et al., 2025). These results suggest that depending on the availability of the malignant transformation can be either promoted or inhibited by the presence of ncRNA partners and readers.

In the same way, opposing effects are observed with the FTO and ALKBH5 erasers. FTO is tumor-suppressive in breast cancer and destabilizes m6A-modified pro-metastatic NEAT1, whereas it promotes the growth of leukemia by demethylating the transcripts of MYC and ASB2 (Qin et al., 2024). ALKBH5 silences pancreatic cancer stemness through decreasing the m6A levels on lncRNA KCNK15-AS1 and promotes the growth of glioblastoma through FOXM1-AS stabilization (He et al., 2021).

Dichotomous functions are also observed in reader proteins. YTHDF1, in some contexts, stimulates translation of transcripts that are immune evasive Like PD-L1, promoting tumor progression, whereas it in other contexts stimulates antigen presentation and the priming of CD8 + T-cells, restraining tumor growth (Han et al., 2021). YTHDF2 breaks down oncogenic transcripts like EGFR in HCC in normoxia, and in hypoxia, orchestrates pro-survival mRNAs and promotes tumor progression.

A significant factor that may add to these context-dependent effects is that tissue-specific expression of ncRNAs searches the spectrum of activity of widespread-expressed m6A regulators. Commonly ubiquitous proteins (METTL3, YTHDF1/2/3, and IGF2BPs) often have diverged functions in different tissues since they interact with distinct ncRNA pools, many of which are developmental restricted and serve either as scaffold, as decoy, as competing substrates. According to the recent profiling research, the competitive environment in which an m6A regulator binds to readers is determined by ncRNA abundance, and can cause an identical regulator to target oncogenic and tumor-suppressive targets according to tissue identity (Zhang H. et al., 2025).

**FIGURE 5:**
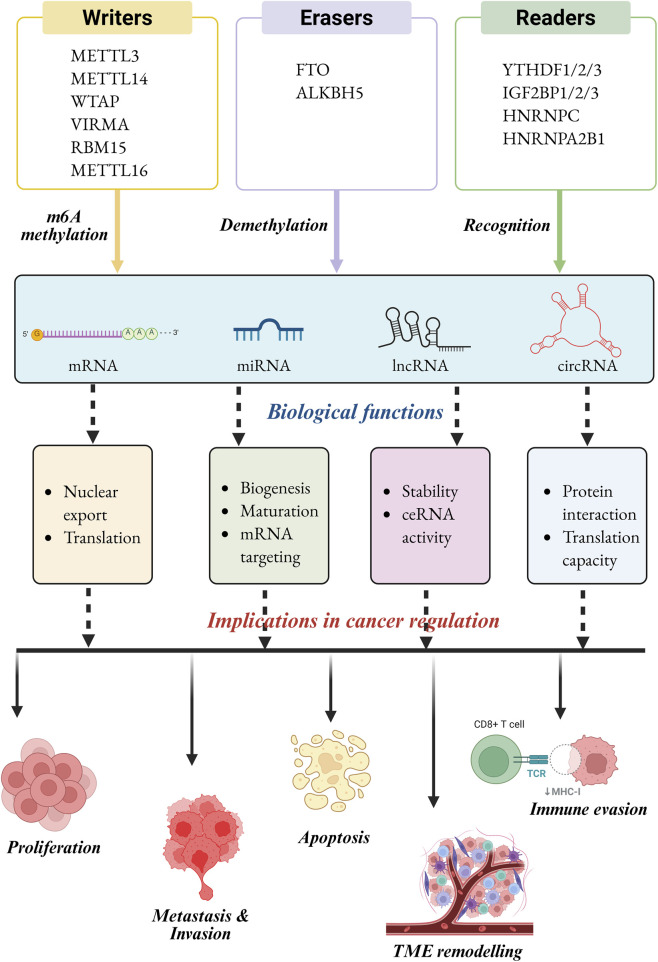
Illustration depicts how m6A writers, erasers, and reader proteins interact with their targets such as mRNA, miRNA, lncRNA and circRNA. The coordinated activities of these factors regulate vital biological processes, including nuclear export and translation, miRNA biogenesis and targeting, lncRNA stability and ceRNA activity, as well as circRNA-mediated protein interactions and translational potential. Further, these functions involve the regulation of cancer *via* cell proliferation, metastasis, immune evasion, apoptosis and remodeling of the tumor microenvironment.

Cumulatively, this information underscores that m6A roles must be viewed in context-specific ncRNA-m6A-reader systems, and there should be no prejudice as to the homogenous, either oncogenic or tumor-suppressive activities of m6A regulators. This understanding on overcoming such contradictions is critical to the development of selective m6A-targeted therapies. Also, the influence of m6A modifications *via* writers, erasers, and readers on the regulation of cancer was depicted in Figure 5.

## 5 Clinical applications of ncRNA-m6A interplay in cancer therapeutic resistance

### 5.1 Role of ncRNA-m6A crosstalk in drug resistance

Research has shown that the interaction between ncRNAs and m6A modification is responsible for drug resistance in many cancers. One study identified that LINC01273 was significantly overexpressed in sorafenib-resistant HCC tissues and was correlated with low survival rates. Knockdown of LINC01273 inhibited cell survival and colony formation in sorafenib-resistant cells, revealing its role in resistance maintenance by miR-600-mediated downregulation of METTL3 (Kong et al., 2022). Another study validated that DUXAP8 contributed to HCC chemoresistance by increasing m6A methylation. Elevated m6A modification stabilized DUXAP8, increasing its expression and facilitating HCC proliferation and migration. DUXAP8 acted as a sponge for miR-584-5p, resulting in upregulation of MAPK1 and activation of the MAPK/ERK pathway, thus maintaining chemoresistance (Liu et al., 2021c). Moreover, in LC, m6A-modified ncRNAs, such as miR-146a and TUSC7, have been found to mediate tyrosine kinase inhibitor (TKI) resistance. In erlotinib-resistant PC9 and HCC827 cells, activation of the miR-146a/Notch pathway by m6A maintained cancer growth and stemness, whereas YTHDF2-mediated repression of TUSC7 promoted resistance *via* a feedback loop involving cMYC and DICER1 (Li K. et al., 2021).

In BC, a study indicated that HNRNPA2/B1, an m6A reader, was involved in endocrine resistance by m6A-dependent regulation of miRNA. Tamoxifen-resistant LCC9 cells overexpressing HNRNPA2/B1 disrupted the miRNAs for progesterone receptor and TGFβ signaling, lowering sensitivity to 4-hydroxytamoxifen and fulvestrant (Klinge et al., 2019). In chronic myeloid leukemia (CML), research indicated that FTO-induced m6A hypomethylation stabilized lncRNAs like SENCR, PROX1-AS1, and LN892, which activated PI3K signaling by upregulating ITGA2, F2R, and COL6A1, hence leading to TKI resistance. Knockdown of such lncRNAs or inhibition of PI3K restored TKI sensitivity, thus making the lncRNA-m6A-PI3K axis a therapeutic target (Liu, 2024). Another study in epithelial OC (EOC) showed that the m6A demethylase ALKBH5 induced cisplatin resistance through the ALKBH5-HOXA10 axis by enhancing JAK2 m6A demethylation and activation of the JAK2/STAT3 pathway. Inhibition of ALKBH5, HOXA10, or JAK2/STAT3 reversed cisplatin sensitivity (Nie et al., 2021). In HCC, circRNA-SORE sustained sorafenib resistance through the promotion of m6A modification. circRNA-SORE acted as a miR-103a-2-5p and miR-660-3p sponge and thereby activated the Wnt/β-catenin pathway to maintain drug resistance. Knockdown of circRNA-SORE initiated sorafenib-induced apoptosis, indicating the potential for drug resistance reversal through targeting m6A-modified ncRNAs (Xu et al., 2020).

### 5.2 Role of ncRNA-m6A interplay in overcoming drug resistance

Modulation of m6A modifications in ncRNAs provides an effective approach to suppress drug resistance in cancer treatment. In TNBC, ZC3H13 downregulation in drug-resistant cells increased doxorubicin (DOX) resistance. Overexpression of ZC3H13 enhanced m6A modification of lncKCNQ1OT1, suppressing its stability *via* YTHDF2. This downregulation interfered with the recruitment of MLL4 to the TRABD promoter, suppressing the expression of TRABD and triggering ferroptosis, which restored DOX sensitivity (Huang et al., 2025). Similarly, in esophageal cancer, high levels of exosomal miR-130a-3p inhibited ferroptosis by inhibiting METTL14-mediated m6A modification of FSP1. Restoration of METTL14 expression reversed the effect, promoting m6A methylation of FSP1 and inducing ferroptosis, thus overcoming cisplatin resistance (Han et al., 2025). Moreover, in pancreatic cancer, METTL3-mediated m6A methylation suppressed lncRNA DBH-AS1, which enhanced gemcitabine sensitivity by sponging miR-3163 and upregulating USP44, ultimately reversing chemoresistance (Ye X. et al., 2022).

In LUAD, metformin promoted DNMT3a/b binding to the METTL3 promoter, increasing m6A methylation at pri-Let-7b. This modification induced Let-7b maturation, inhibited cancer SCs’ self-renewal and Notch signaling, and restored TKI sensitivity (Li K. et al., 2022). Additionally, circFBXW7 also played a role in evading osimertinib resistance in LUAD by blocking Wnt signaling. YTHDF3 facilitated the m6A-dependent translation of circFBXW7 into the polypeptide circFBXW7-185AA, which interacted with β-catenin, promoting its ubiquitination and degradation. This inhibited Wnt signaling, reducing cancer SCs’ renewal and resistance (Li et al., 2023a). In another study on LUAD, FENDRR downregulation was linked to cisplatin resistance. METTL3-mediated m6A modification facilitated FENDRR degradation through YTHDF2, while FENDRR overexpression increased TFRC expression by sponging miR-761, enhancing ferroptosis and restoring cisplatin sensitivity (Zhao et al., 2025).

### 5.3 Pharmacological targeting of m6A machinery: inhibitors and translational status

Pharmacological control of m6A regulators is also an approach that is becoming amenable to breaking oncogenic m6A -ncRNA interactions. The latest most advanced writer-targeting agents are METTL3 inhibitors. STM2457 is a first-in-class SAM-competitive METTL3 catalytic inhibitor with potent anti-leukemic and anti-tumor effects in a variety of preclinical models through the suppression of m6A on oncogenic mRNAs and ncRNAs (Yuan F. et al., 2025). Still more recently, the first METTL3-inhibitor in human case studies was the STC-15 (oral), which is in phase-1 dose-escalation/expansion in solid tumors, with interim clinical safety/PK/PD reported in 2024 (Moser et al., 2024). Other preclinical METTL3 inhibitors (e.g., UZH1a and analogs) are in progress of pre-clinical optimization in terms of selectivity and tumor delivery (Wu et al., 2024).

FTO inhibitors are widely preclinically validated among the group of so-called eraser-targeting drugs. Rhein and meclofenamic acid were the first to demonstrate proof-of-concept (Zhou et al., 2021) and more potent compounds FB23/FB23-2 and recent high-potency inhibitors CS1/CS2 suppress AML and some solid tumor m6A-dependent degradation of oncogenic transcripts and related ncRNAs (Huang et al., 2019; Gao et al., 2024). Moreover, selective ALKBH5 inhibitors are in their infancy growing at a pace. Preclinical tests using covalent and non-covalent tool compounds including TD19 and pyrazole-pyrimidine derivatives demonstrate cell-active ALKBH5 inhibitory activity with anti-AML/anti-GBM activity, which proves ALKBH5 as a drug target to reverse m6A-stabilized oncogenic RNAs and ncRNAs (Zhang X. et al., 2025; Lai et al., 2024).

Comprehensively, these agents demonstrate the capacity of translation between studies: inhibiting writer (METTL3) is more likely to repress m6A-supported oncogenic networks of translation/processing, but inhibition of erasers (FTO/ALKBH5) restores m6A marks that direct malignant RNAs toward degradation. The tumor-selective deployment will have to be implemented with the help of ncRNA-m6A signatures to achieve success in the future.

**FIGURE 6:**
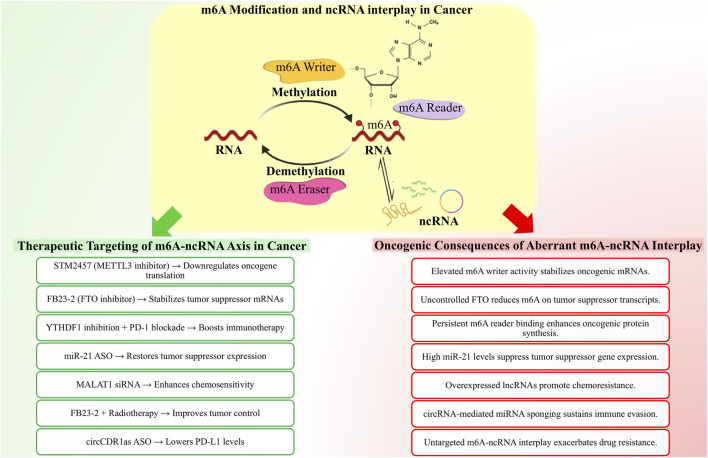
An overview of m6A modification (*via* writers, erasers, and reader) and ncRNA interplay in cancer was depicted.

Even though a few METTL3 and FTO/ALKBH5 inhibitors present a potentially positive antitumor response, their developmental relevance in a translational model is critically reliant on efficient and targeted delivery. Recent evidence shows that lipid nanoparticles, polymer-based carriers and engineered extracellular vesicles can increase the level of intracellular accumulating of m6A-modulating agents and decrease clearance, can be used to promote pharmacodynamic exposure of tumor tissues (Ma et al., 2023; Yan Y. et al., 2025). Nonetheless, small-molecule inhibitors present undesirable off-target effects (Chen Q. et al., 2020), such as changes in RNA stability, immune responses, and unintended impacts on the hematopoietic tissue, or neural tissue, due to the thousands of transcripts regulated by m6A machinery (Lightfoot and Smith, 2025). Focused delivery *via* tumor-specific LNPs or antibodies drug conjugates become necessary to reduce toxicity. In addition, catalytic turnover of METTL3 is sluggish and the concentration amount of FTO in cells is high, posing difficulties to sustain target engagement, necessitating prolonged intracellular residence time inhibitors, and low cross-reactivity of other Fe(II)/αKG dioxygenases (Du et al., 2021). These reflections highlight the fact that pharmacologic m6A modulation must be enzyme selective as well as delivered, ensuring delivery of such drugs result in therapeutic levels without systemic disruptive effects on epitranscriptome. An overview of m6A modification and ncRNA interplay in cancer was depicted in Figure 6.

## 6 Challenges and future directions

Although the study of m6A modification in cancer therapy is fast growing, it is accompanied by various difficulties, especially its controlling ncRNAs. Major challenges to be overcome to fully utilize m6A alteration are knowledge of the intricate regulatory networks involving m6A, development of accurate tools for its investigation, and application of these insights into clinical settings (Qi et al., 2023; Tooley et al., 2023).

The intricacy of m6A control presents one of the toughest difficulties facing the sector. Under mediation by “writers,” such as METTL3 and METTL14, “erasers,” such as FTO and ALKBH5, and “readers,” such as YTHDF proteins, m6A modification is a dynamic, reversible process (Huang et al., 2020; Qi et al., 2023). These enzymes add, delete, and identify m6A modifications on RNA molecules, working in concert, therefore affecting their stability, splicing, translation, and destruction. Demonstrating how m6A alteration affects cancer development is the way METTL3 enhances the maturation of carcinogenic miRNAs (Chen Y. et al., 2020; Ma et al., 2019). For pancreatic ductal adenocarcinoma (PDAC), for instance, METTL3 targets tumor suppressor genes, increasing the miR-25-3p production and so fostering carcinogenesis. METTL3 similarly helps miR-1246 to mature in CRC, involved in cancer metastases (Chen and Wong, 2020). Conversely, YTHDF proteins such as YTHDF1 and YTHDF2 bind to m6A-modified mRNAs, thereby either boosting translation or destruction, respectively (Wang et al., 2020; Zhou Z. et al., 2020). The double use of m6A complicates the research of its function in cancer since the same m6A change might have different effects depending on the type of cell and environment. For example, it has been demonstrated how m6A-modified mRNAs broken down by YTHDF2 affect liver cancer cell sensitivity to chemotherapeutic agents (Lin H. et al., 2022; Tang et al., 2024; Liu F. et al., 2024). Treatments aiming to target m6A rely on an awareness of how these enzymes interact to modify the cancer transcriptome in a tissue- and context-specific way. The absence of exact techniques for analyzing m6A changes, especially *in vivo*, presents still another great obstacle (Yao et al., 2022; Porto et al., 2019). There is also another limitation that is due to specificity and affinity of popular m6A antibodies that directly affect the interpretation of MeRIP-seq and m6A-seq data. Peak profiles that result from variation between batches and cross-reactivity with structurally related adenosine substitutions can also be different. Empirical comparisons done by benchmarking commercial antibodies indicate that signal strength generally is correlated with abundance of transcripts and is not necessarily the actual stoichiometry of methylation and thus requires orthogonal validation e.g., miCLIP, SCARLET, or direct RNA sequencing to validate m6A-sites (McIntyre et al., 2020; Ye H. et al., 2024; Körtel et al., 2021).

Methods including m6A-sequencing (m6A-seq) have given important new perspectives on the worldwide m6A modification distribution over the transcriptome. M6A-seq does not, however, adequately capture the real-time dynamics of m6A alteration during events like cancer growth, so its resolution is limited (Hu et al., 2023). Moreover, m6A-seq cannot evaluate the precise timing of modifications or distinguish between several m6A variants on the same RNA species. The availability of m6A-specific antibodies represents still another restriction (Lou et al., 2024b). Studying the exact functional ramifications of m6A *in vivo* is challenging since many antibodies lack the specificity needed to precisely estimate m6A levels in certain RNA molecules. New approaches, including CRISPR-based tools, which let m6A dynamics be directly visualized, are being developed to help overcome these obstacles. For GBM cells, for example, METTL3 has been deleted *via* CRISPR interference (CRISPRi), therefore exposing its vital function in tumor growth (Ye D. et al., 2022; Yi et al., 2023). Furthermore, deletions of FTO brought by CRISPR-Cas9 in leukemia cells revealed that, *via* changing the expression of cell-cycle regulators, the elimination of this m6A eraser dramatically slows down cancer cell proliferation (Seimiya et al., 2020). Although these CRISPR-based technologies provide a more exact approach to investigating m6A modification, additional development of real-time monitoring tools is necessary to grasp how m6A modification alters throughout cancer growth (Hussen et al., 2023; Zhang S. et al., 2025).

Another exciting path, but laden with difficulties, is the therapeutic targeting of m6A regulators. Crucially, in control of oncogenes and tumor suppressor expression is m6A-modifying enzymes, including METTL3, METTL14, and FTO (Alam and Giri, 2024). In BC, METTL3 controls the expression of oncogenic miR-25-3p; in GC, METTL14 has been demonstrated to decrease metastases by influencing the maturation of miR-126, an anti-oncogenic miRNA (Zhang et al., 2024b). While stopping tumor growth by blocking m6A “writers,” including METTL3 or METTL14, this strategy is confounded by the tissue-specific roles these enzymes play in other processes, including immune response control or hematopoiesis (Chen et al., 2022e). Conversely, by demethylating tumor suppressor genes like p53, the m6A eraser FTO has been linked to aggravating the spread of many malignancies, including GBM. In preclinical models of leukemia and GBM, where it decreased tumor cell growth and improved chemotherapy efficacy, inhibition of FTO has shown interesting outcomes (Condic et al., 2022). Still, the specificity of these inhibitors is a big issue since they might also interfere with regular cellular operations and cause side effects (Su et al., 2025). The difficulty is in creating inhibitors that specifically target m6A-modifying enzymes in cancer cells without compromising their functions in regular cellular operations. Moreover, the diverse purposes of these enzymes in different tumors make it difficult to create universal treatments aiming at m6A regulators throughout several cancer types (Ralser et al., 2023).

The bidirectional control between m6A and ncRNAs adds still another level of intricacy that hampers the research on the function of m6A in cancer. Although m6A modification controls ncRNAs’ expression, new research has revealed that ncRNAs themselves can also influence m6A regulators’ expression, hence forming a feedback loop (Zhang N. et al., 2021). LncRNA DANCR, for instance, has been demonstrated to upregulate METTL3 expression, hence enhancing m6A modification of target RNAs and encouraging tumor growth in GC (Jiang J. et al., 2024). In addition to complicating the regulatory network, the back loop between ncRNAs and m6A regulators creates novel therapeutic opportunities. One fresh treatment approach could be aiming to target the ncRNAs controlling m6A (Cusenza et al., 2023). By silencing lncRNAs that support METTL3 expression, m6A-modified RNAs oncogenic potential can be lowered, and this feedback loop in cancer cells can be effectively broken. Given that their roles vary depending on the kind of cancer and stage of tumor progression, it remains difficult to estimate the importance of ncRNAs involved in controlling m6A writers and erasers (Forrest and Khalil, 2017). At last, the tumor heterogeneity seen across cancer patients impedes the clinical application of m6A-based cancer treatments (Herrera-Solorio et al., 2017). At the molecular level, cancer is quite diverse; hence, the expression of m6A regulators and ncRNAs might vary greatly between patients even inside the same tumor type. For example, whereas in other malignancies, such CRC, METTL3 overexpression is linked with poor survival outcomes; HCC, METTL14 expression correlates with improved prognosis (Dlamini et al., 2024). This requires the creation of tailored treatments considering the m6A modification pattern and ncRNA profile of every tumor. Personalized treatment plans might be guided by biomarkers reflecting m6A alteration and ncRNA expression in cancers (Russo et al., 2018). Moreover, combining m6A-targeted treatments with other modalities, such as checkpoint inhibitors or chemotherapy, could have synergistic effects, so boost therapeutic efficacy and overcome drug resistance. Further studies are required, nevertheless, to evaluate the safety and effectiveness of these mixed treatments (Wang X. et al., 2022; Chan and Tay, 2018).

## 7 Conclusion

m6A modification shows dynamic reversible characteristics that represent a key epitranscriptomic regulatory function which affects RNA metabolism, together with its stability and translation, and subsequent degradation. Multiple ncRNAs-derived fragments partner with m6A modifications to determine the oncogenic landscape, which provides new opportunities for cancer therapeutic advances. The regulation of oncogenes and tumor suppressor RNAs through m6A modifications occurs within a wide range of contexts because reader proteins, including YTHDF1/2/3 and IGF2BP family members, determine RNA fate, which leads to changes in cancer progression.

In addition to their actions on m6A regulators, ncRNAs activate reciprocal effects on erasers (FTO, ALKBH5), writers (METTL3, METTL14), and readers, which leads to intricate feedback networks that either boost tumorigenic characteristics or present therapeutic possibilities. The current findings integration demonstrates that ncRNA-m6A interaction mediates cancer progression and major clinical treatment hurdles such as therapy resistance, tumor cell spreading and immune system avoidance, and self-renewing potential maintenance.

The challenge exists in successfully developing clinical applications from discovered molecular information. The heterogeneity between tumors and opposing effects of m6A machinery and dynamic ncRNA patterns force healthcare providers to adopt individualized therapeutic measures. Novel CRISPR epitranscriptome editing methods alongside small molecule m6A inhibitor developments, along with RNA therapeutic innovations, offer hopeful potential but need additional improvements in execution precision and optimal delivery systems to prevent undesired side effects.

Research needs to focus on combining different types of omics data to construct m6A and ncRNA networks among different cancer-related environments and patient demographics. It is crucial to develop precise systems that monitor m6A modifications in real time inside living systems. The most effective approach to fight cancer resistance will incorporate individualized combination therapies that unite m6A modulating agents and ncRNA-focused interventions and established therapeutic approaches to achieve sustained cancer elimination.

Our knowledge expansion of the epitranscriptomic-ncRNA interaction axis brings us nearer to extracting its maximal therapeutic value, which leads to improved cancer treatment options with enhanced precision and adaptability.
